# Laser-induced electron diffraction: Imaging of a single gas-phase molecular structure with one of its own electrons

**DOI:** 10.1063/4.0000237

**Published:** 2024-08-30

**Authors:** K. Chirvi, J. Biegert

**Affiliations:** 1 ICFO—Institut de Ciencies Fotoniques, The Barcelona Institute of Science and Technology, 08860 Castelldefels (Barcelona), Spain; 2 ICREA, Pg. Lluís Companys 23, 08010 Barcelona, Spain

## Abstract

Among the many methods to image molecular structure, laser-induced electron diffraction (LIED) can image a single gas-phase molecule by locating all of a molecule's atoms in space and time. The method is based on attosecond electron recollision driven by a laser field and can reach attosecond temporal resolution. Implementation with a mid-IR laser and cold-target recoil ion-momentum spectroscopy, single molecules are measured with picometer resolution due to the keV electron impact energy without ensemble averaging or the need for molecular orientation. Nowadays, the method has evolved to detect single complex and chiral molecular structures in 3D. The review will touch on the various methods to discuss the implementations of LIED toward single-molecule imaging and complement the discussions with noteworthy experimental findings in the field.

## I. INTRODUCTION

One of the grand challenges in science is visualizing how a molecule undergoes a structural transformation, such as a conformational change to bond with another molecule, or how it isomerizes. Imaging ideally requires pinpointing the location of all the atoms that constitute the molecule in space and to track them over time. One of the many challenges toward such a goal is isolating a single molecular (MO) system in order not to average over random orientations or conformations while still being able to detect all its atoms. Molecular alignment^1^ or orientation may be a remedy, but it depending on the specific molecular system. Imaging with electrons^2^ rather than photons^3,4^ has the advantage of vastly increased scattering cross section, consequently a much higher sensitivity, and a much higher resolution due to the picometer de Broglie wavelength, but it comes at the cost of Coulomb repulsion which mainly limits the temporal resolution. State-of-the-art gas-phase ultrafast electron diffraction (UED) utilizes a high-energy electron pulse, which is generated by an optical UV pulse by extracting electrons from a photocathode and accelerating them to energies in the range of hundreds of keV thus, achieving sub-Ångstrom spatial resolution.^5,6^ One challenge for UED is achieving sufficient electron beam brightness to achieve an adequate detection signal. This typically requires thousands of electrons^7^ whose Coulomb repulsion limits the temporal resolution to about 100 fs.^8^ For molecular imaging, another challenge is achieving the scattering signal, which requires sufficient target density due to the incoherent electron scattering process, and, consequently, single-molecule imaging has not been achieved.^2,9^ These limitations have spurred numerous developments^2,8,10–15^ to remedy the intrinsic limitations of electron diffraction imaging^16^ through some sort of electron phase space manipulation to compress the electron bunch^17,18^ or by using relativistic energies.^8,19–21^ For instance, a carrier envelope phase (CEP)-stable strong optical field led to an electron bunch structured into a train of 15 as pulses^22^ and nanostructures^11,23,24^ have been used to confine the duration to 53 as.^25^ A very recent implementation of terahertz streaking with a field strength of 100 kV/cm inside a UED setup demonstrated compression to 6 fs duration.^26^ Another interesting variant of UED that achieves few-femtosecond temporal resolution is laser-assisted electron diffraction (LAED).^27,28^ In this approach, electrons with keV-level energies interact with a molecular ensemble while an external femtosecond light pulse modifies the kinetic energy of the scattered electrons, thus effectively acting as an ultrafast optical gate; thus, the temporal resolution is largely determined by the laser's pulse duration. Neither method has achieved single-molecule imaging. Here, we focus on laser-induced electron diffraction (LIED),^29,30^ which is based on coherent self-imaging with an elastically rescattering attosecond electron wave packet (EWP).^29,31–36^ The coherence of the self-imaging process is the key difference of LIED to the before-mentioned methods as it provides sensitivity for single-molecule imaging. Once the conditions for achieving “hard” collisions are fulfilled to pinpoint the location of the atomic constituents of a molecular structure and sufficient momentum transfer is achieved to resolve them, the advantage of such a “molecular selfie” is that the method intrinsically achieves the physical limits of space and time imaging (picometer and attosecond).^31,35,37^ As with any method, one has to be mindful of the caveats, one of them being that tunnel ionization intrinsically starts dynamics. However, we, and others, have shown that in most cases, this is no detriment to molecular imaging and to studying molecular structure.^38^ As for any scattering method, the achievable maximal momentum transfer determines the spatial resolution.^39^ Conventional electron diffraction (CED) achieves this for small angle scattering through high electron impact energies; for instance, Ihee *et al.*^40^ achieved a momentum transfer of up to 18.5 Å^−1^ for angles less than 11.3° with electrons reaching 30 keV. Such impact energies are unattainable with strong-field electron rescattering as the laser intensities would be so high that the ejected electron would not return to its target and scatter. However, Xu *et al.*^7^ have shown that a comparable momentum transfer of 16 Å^−1^ can be achieved for much more moderate impact energies of 300 eV for backward scattering.^7,41^ Before we describe the evolution of LIED and the various implementations, we discuss the underlying physical mechanism and relevant aspects important for LIED.

## II. THE BASIS FOR LASER-INDUCED ELECTRON DIFFRACTION (LIED)—STRONG-FIELD ELECTRON RECOLLISION

Within the very large body of work^42–52^ describing electron recollision in a strong laser field, we focus on aspects relevant for LIED to achieve accurate measurement of the location of the atomic constituents of a molecule. To fulfill this goal, several, partially interrelating, conditions have to be met:

(1) *The rescattering electron needs to be released through tunnel ionization from the target.* The tunneling or quasi-static ionization condition ensures sufficiently small initial momentum of the rescattering electron (classically the velocity of the tunneling electron is zero) such that the measured field-dressed scattering momenta can be transferred into field-free scattering momenta by subtraction of the laser vector potential.

In his seminal work on strong-field ionization, Keldysh^61^ introduced the dimensionless adiabaticity parameter $\gamma ≔\omega \sqrt{2m_{e}\;I_{p}}/\left(eE\right)$, which can be expressed in terms of the ponderomotive energy $U_{P}=e^{2}E^{2}/\left(4m_{e}\omega^{2}\right)$ as $\gamma ≔\sqrt{I_{P}/\left(2U_{P}\right)}$ and distinguishes between the two main regimes of ionization, i.e., the multiphoton ionization (MPI) ( γ>1) and the tunneling ionization (TI) or quasi-static ionization regime for γ≪1. It is important to realize that γ should only be used as an approximate orientation to determine whether a process is predominantly governed by MPI or TI. It was early on pointed out by Reiss^54–58^ in a series of publications that the adiabaticity parameter alone is insufficient to determine the boundaries of the TI and he introduced two further parameters that have to be taken together to determine the exact regime of ionization. The introduced parameter $z_{1}≔1/\gamma^{2}$ determines the relation between the ponderomotive energy and the ionization potential. $z_{f}≔2U_{P}/\left(m_{e}c^{2}\right)$^59^ determines whether the ponderomotive energy is comparable to the rest kinetic energy and $z≔U_{P}/\omega$ provides a measure of extend of relativistic contribution.^55^ Thus, TI requires nonlinear ionization $\omega \ll I_{P}$ and γ≪1 in addition to the validity of the dipole approximation z≪2c and nonrelativistic behavior $z_{f}\ll 1$. Applying these criteria, it is important to recognize that most strong-field and some LIED experiments were conducted in a mixed MPI and TI regime in the dipole regime with consequent difficulties for a clear interpretation or measurement result. The only viable solution to achieve electron recollision for low I_P_ targets such as molecules is the increase in wavelength while keeping the intensity moderate.^50^ Among the many models to describe ionization, Nikishov and Ritus^59^ computed transition rates for the interaction between an electromagnetic wave and atomic systems composed of short-range potential barriers. The Keldysh–Faisal–Reiss (KFR) theory^60–62^ stands as a significant advancement in our comprehension of photoionization amidst the presence of an intense laser field, while the strong-field approximation (SFA) by Faisal and Reiss offers a practical implementation by disregarding the atom's Coulomb field post-ionization. The introduction of the PPT model by Perelomov, Popov, and Terent'ev^53^ marks another milestone, as it incorporates Coulomb forces to estimate tunneling transition rates. Ammosov, Delone, and Krainov (ADK)^63^ developed an expression for ionization rates in the quasi-static regime and for atoms beyond hydrogen. The ADK model is widely used, but the PPT model generally results in a more accurate description of the ionization process, see, e.g., Ref. 64. Thus, Popruzhenko *et al.*^65^ introduced an extended PPT model which describes atomic photoionization independent of adiabaticity parameter and for a large range of laser intensities in very good agreement with *ab initio* calculations. Turning to molecular systems, Tong *et al.*^66^ proposed the molecular ADK theory (MO-ADK) to account for additional degrees of freedom, such as rotational and vibrational motion, as well as the non-spherical electrostatic potential. Furthermore, with improved experimental methods, it was found that the long-range Coulomb potential can have a pivotal impact on the electron and on its final state. Experimental investigations revealed the so-called low- and zero-energy structures (LES and ZES)^67–69^ in the photoelectron momentum distribution. These findings triggered new work to better incorporate Coulomb effects^70,71^ and it was found that the effect of the Coulomb potential is significantly reduced at mid-IR wavelengths compared to shorter wavelengths of 800 nm.^72,73^ Belsa *et al.*^74^ quantified the error due to neglecting the Coulomb potential by employing a comparison between classical recollision model, a classical trajectory Monte Carlo (CTMC) simulation including the Coulomb potential, and compared with full quantum dynamics simulations based on the time-dependent Schrödinger equation (TDSE). They found that the error in return energy due to Coulomb effects diminishes to a negligible ∼0.5% leading to worst-case bond retrieval errors of 5% at 3.2 *μ*m compared to 11% at 2 *μ*m and 4% at 4 *μ*m.

(2) *The rescattering electron needs to return with high impact energy to achieve a core-penetrating collision.* The de Broglie wavelength of the scattering electron needs to be small enough to resolve atomic distances, its spatial extent needs to be sufficient to cover the molecule, its waveform should approximate a plane wave, and its momentum needs to be high enough not avoid deflection on the valence electron cloud to get deflected on atomic cores.

An essential part of the ionization process in strong laser fields is the propagation of the electron wave packet after tunneling and its possibility for rescattering. The possibility of a return and interaction of the tunnel electron with its parent ion was first suggested by Kuchiev^75^ and further discussed in Refs. 76–78. Corkum formulated the idea of a purely classically electron return,^79^ nowadays known as the recollision, three step, or simple man's model (SMM). Its validity, aspects, and deviations are discussed in the large body of literature.^36,48,80–85^ Here, we refer again to the finding that the SMM is very well justified in the TI for intensities below the saturation limit, which scales favorably with long wavelength.^65^ Another significant advantage of utilizing long wavelengths is the quadratic relationship between the ponderomotive energy and wavelength. Consequently, increasing the wavelength of the driving laser leads to a quadratic increase in the energy of the re-colliding electron, as predicted by the SMM. This correlation can be explained by the extended duration over which an electron experiences acceleration in the electric field throughout a longer optical cycle, both away from and toward the parent ion. A comparison between 0.8 and 3 *μ*m reveals a substantial increase in return energy by a factor of 14, i.e., for a peak intensity of 10^14^ W/cm^2^, from U_P_ = 18 eV at 0.8 *μ*m to 252 eV at 3 *μ*m. Sanchez *et al.*^86^ achieved an electron return energy of 285 eV resulting in backscattered electron kinetic energies of ∼1 keV. Additionally, longer wavelengths offer another benefit, particularly for large molecules, since ionization potential typically decreases with target size. They help mitigate excessive multiple ionization of molecules by using moderate intensities.

An important aspect that is amply discussed in the literature^87–89^ is the existence of multiple quantum trajectories and multiple returns for a multi-cycle laser field. Quantum mechanically, given return energy can be achieved by two different trajectories ionizing and returning at distinct times. These trajectories are called “long” and “short,” and the terminology refers to the propagation time in the continuum. That is, the long trajectory electron is ionized earlier and returns later in the laser cycle compared with the short trajectory electron. It is well established that the long trajectory dominates (10^4^ times) over the short trajectory since it is launched close to the field maximum with correspondingly much higher ionization yield. This dominance reduces to unity at the maximum return energy. We like to note that the common misconception that the short trajectory dominates in LIED stems from referring to high harmonic generation (HHG) in which the phase-matching process from a vast array of emitters favors the short trajectory contribution.^90,91^ However, on the single atom or single-molecule level, the long trajectory dominates. This condition sets the stage for the reconstruction of field-free scattering momenta from the measured field-dressed scattering momenta. Another important aspect for many-cycle laser fields is multiple returns. We note that the general aspect of multiple returns can be understood with the SMM, and their exact behavior and trajectories require Coulomb corrected models or *ab initio* methods. Furthermore, we note that increasing the wavelength from near- to mid-IR increases the accuracy of the SMM predictions for the trajectories.^92,93^ In essence, the tunneling electron can re-scatter multiple times in a many-cycle laser field with the 1^st^ return reaching a maximum impact energy of 3.16 U_P_. There exists however the possibility that the electron re-scatters and returns a second time in the successive laser cycle with maximum energy of ∼1.6 U_P_. The process can occur another time, and the electron returns a third time with an even higher energy of ∼2.5 U_P_.^72^ Out of all these possibilities, it suffices to know that the 3^rd^ return is the second most energetic one and its impact on LIED continues to promote debates that this can be problematic. Let us first realize what the 3^rd^ return signifies. A simple SMM calculation tells us that the long trajectory at 2.5 U_P_ impact energy leads to a maximum rescattering energy of 8.6 U_P_. A remedy would be to restrict the entire data analysis in LIED to the range 8.6–10 U_P_. Obviously, the yield of re-scattered electrons close to the maximum classical cutoff of 10 U_P_ scales unfavorably with $\lambda^{-\left(4-5\right)}.$^94^ Consequently, the proportion of re-scattered electrons (with kinetic energy greater than 2 U_P_) to direct electrons (with kinetic energy less than 2 U_P_) varies by a factor of 200 when comparing driving wavelengths of 0.8 and 3.0 *μ*m. In practical terms, transitioning from near- to mid-IR wavelengths for laser experiments extends the duration of an experiment by 200 times, assuming all other laser parameters remain constant. One potential approach to mitigate this increase in duration is to increase the repetition rate of the laser system. Another remedy to entirely remove this issue is to conduct the LIED measurement with a sub-2-cycle laser pulse or with a two-color field.^95^ Even if not restricted to this range, the worst-case error in return time would be one optical cycle, e.g., at 3 *μ*m increasing the time of return from 9 to 20 fs for rescattering energies below 8.6 U_P_, thus potentially averaging over effects such as the onset of a vibration or bending.

Another aspect of electron recollision is the release of a secondary electrons, termed double ionization (DI), which occurs sequentially (SDI) or non-sequentially (NSDI).^44–52,96–99^ The DI process is intricately linked to the recollision of the first electron and mainly proceeds via two routes: the second electron can either be directly ionized via the so-called (e, 2e) mechanism or it can be resonantly excited and subsequently tunnel ionized at a later time (RESI).^100,101^ Double ionization is a complex process that is known to be dependent on a number of laser parameters such as the intensity, polarization, pulse duration, and wavelength and the LIED experiment needs to be checked to avoid such contributions. Since the regime in which single-electron rescattering (LIED) dominates is close to the regime at which DI occurs, it is imperative to check that DI is not prevalent since it would influence the accuracy of the measurement. Thus, detection methods that use ensemble averaging or that cannot distinguish whether doubly charged cations, and whether several electrons are measured, need to find remedies to avoid this problem. As we will explain below, our specific methodology of single-molecule detection in full kinematic electron–ion coincidence with a reaction microscope (REMI) avoids such problems since it detects the existence of a second electron or a doubly charged cation at single shot and automatically discards the counts. The single electron–ion coincidence detection further enables to apply a filter of the electron signals according to experimental conditions such as laser intensity and pulse duration, or selection on momenta ranges to ensure “identical” implementations within a certain limit. Fluctuations or drift over the measurement can be eliminated and data quality can be increased. Finally, we like to touch briefly on another beneficial aspect of electron recollision in long-wavelength fields which is the wave packet spread and its curvature due to quantum diffusion.

The width of the returning electron wave packet (EWP) depends on serval factors, including the initial momentum space distribution of the electron at the tunnel exit,^102^ propagation time,^79,84,103^ and effects of Coulomb focusing^104^ during propagation. These phenomena have been extensively scrutinized.^105^ Neglecting Coulomb focusing, the spreading of the EWP during propagation scales approximately linearly with the drive's wavelength; i.e., longer wavelengths achieve a much planar wavefront. To understand the magnitude of a possible error due to wavefront curvature of the returning wave packet (RWP),^106^ we compare the EWP width $\Delta x_{1/e}$ for a path difference of 180° to the spatial extent of the molecule's atomic arrangement d. For instance, a simple calculation shows that $\Delta x_{1/e}\approx 150\;Å$ for a 3 *μ*m field.^107^ Let us contrast this with the spatial extend of a larger molecule, say fenchone (C_10_H_16_O), whose spatial atomic extent is 6.4 Å. The simple comparison clearly shows that curvature of the wavefront is entirely negligible with respect to the spatial atomic extent of the molecule. How does this scale with wavelength of the laser for driving LIED? An estimation shows that to cover a 30 Å transverse area, a 3 *μ*m field results in a curvature of ∼1.3 Å across the transverse extent while an 800 nm wavelength results in a curvature of 6 Å. Hence, longer wavelength drivers support returning wave packets with close-to-plane wave fronts, further reducing the effective variance of the measurement.

(3) *The core-penetrating rescattering electron needs to achieve sufficient momentum transfer and the measured field-dressed doubly differential scattering cross section (DCS) needs to be converted to a field-free cross section*. Sufficient momentum transfer is achieved by a combination of high impact energy and large angle scattering, reaching its maximum for back scattering.

The field-free differential cross section can be extracted from the field-dressed measurement if the vector potential is known. LIED is readily understood in the framework of the quantitative rescattering theory (QRS),^108^ which is valid in the TI regime and shows that the influence of the intense laser field is separable from the electron/ion back scattering process. For a detailed discussion on the QRS model, we refer to Refs.^108^ and 114 . When impact energies are sufficiently high compared to the binding potential, i.e., above 50 eV, the atomic core is predominantly responsible for electron scattering at large angles rendering the binding orbitals essentially transparent.^7,33,109^ Hence, the independent atom model (IAM)^110^ provides bond distances from the field-free DCS. It is important to emphasize the significance of a high momentum transfer range for such analyses. For instance, electron scattering factors of neutral and charged atoms can differ significantly for momentum transfer below 1.3 Å^−1^. However, once the momentum transfer exceeds 2.5 Å^−1^, these scattering factors become essentially indistinguishable.^111^ We note that it is challenging to attain LIED conditions for the validity of the QRS theory with wavelengths below ∼1.5 *μ*m at the required moderate U_P_. Nevertheless, the conditions are readily satisfied by wavelengths in the longer (mid-IR) wavelength range. Another important benefit of LIED compared to CED is the sensitivity to scatterers whose near-zero cross section at >keV energies make detection impossible. An example is hydrogen, which is ubiquitous in molecular and biochemical reactions. Wolter *et al.*^35^ have shown that LIED is indeed sensitive to proton motion due to the significant DCS at 50 eV and Liu *et al.*^112^ have used the sensitivity to image water molecules.

**FIG. 1.**
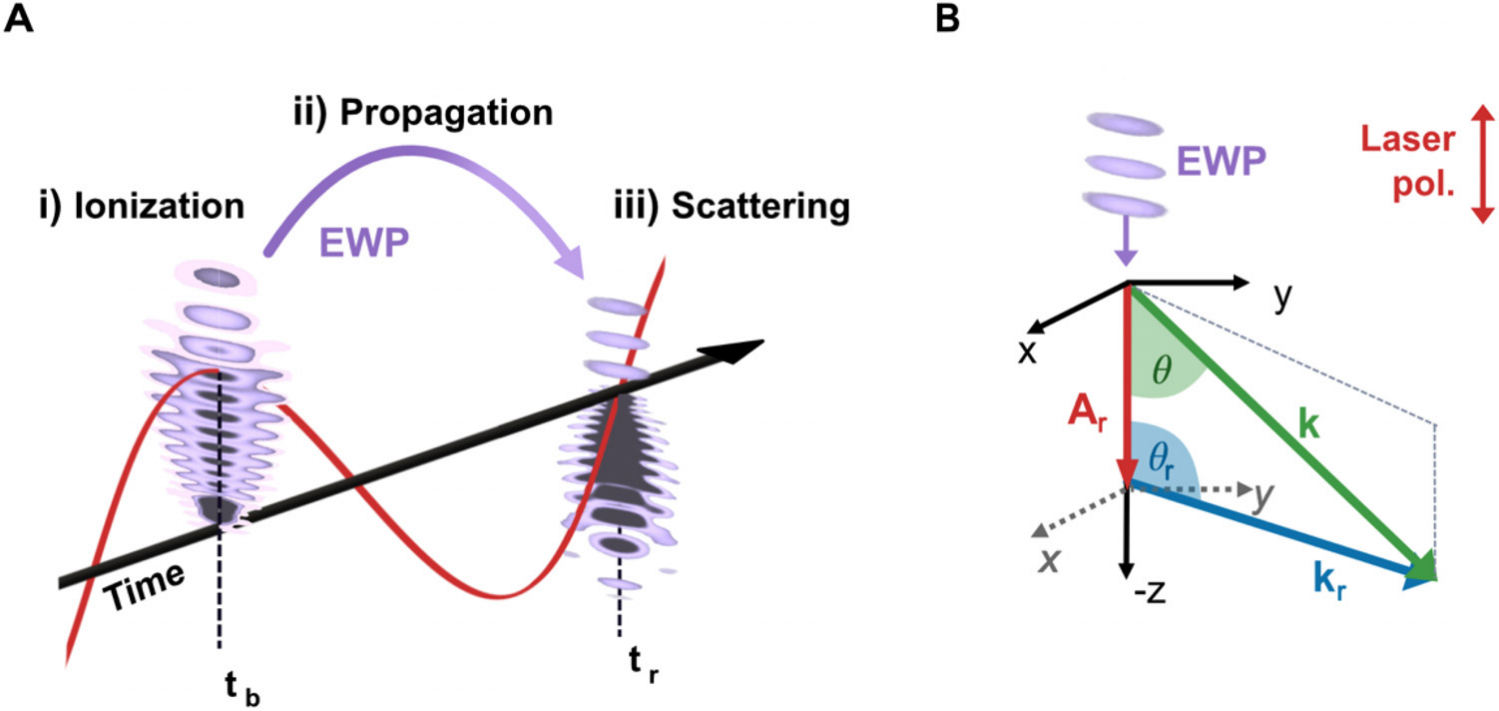
(a**)** Laser-induced electron diffraction is explained in three steps: (i) Tunnel ionization of the molecule at the time of ionization (birth) *t_b_*, (ii) propagation of the electron wave packet (EWP) within the laser field, and (iii) scattering of the EWP at the electron's return time *t_r_.* (b) The measured electron's momentum, *k* (green arrow), is defined in the laboratory frame (black solid coordinate system). In contrast, the returning momentum *k_r_* (blue arrow) and the scattering angle $\theta_{r}$ of the electrons are given in the laser polarization frame (dashed coordinates).

According to the QRS theory, the photoelectron momentum distribution $D\left(k,\theta \right)$ as a function of the final, detected momentum *k* and the final, detected scattering angle θ is linked to the elastic DCS $\sigma \left(k_{r},\theta_{r}\right)$ as a function of returning momentum $k_{r}$ and rescattering angle $\theta_{r}$ by the formula $D\left(k,\theta \right)=W\left(k_{r}\right)\sigma \left(k_{r},\theta_{r}\right),$ where $W\left(k_{r}\right)$ represents the returning electron wave packet (RWP) and $W\left(k_{r},\theta_{r}\right)=\omega_{TI}\left(\theta_{L}\right)\;\tilde{W}\left(k_{r}\right)$, where $\omega_{TI}\left(\theta_{L}\right)$ is the TI rate of the molecule. The elastic DCS $\sigma \left(k_{r},\theta_{r}\right)$ can be computed using standard potential scattering theory (cf. Ref. 113), wherein the elastic scattering of an electron on a spherical potential *V(r)* is solved via the time-independent Schrödinger equation (TISE). A comprehensive overview of this calculation is provided by Chen *et al.*,^108^ resulting in the elastic DCS as the squared sum of scattering amplitudes. By a computation of the EWP with SFA theory and for rescattering simulations using the TDSE, the authors of Refs. 108 and 114 found that SFA accurately describes the EWP dynamics under TI conditions. Both simulations demonstrate that $W\left(k_{r}\right)$ is unaffected by $\theta_{r}$, implying that $W\left(k_{r}\right)$ behaves akin to a beam of incident electrons with momentum $k_{r}$. A similar dependency on $k_{r}$ has been observed, and the shape of $W\left(k_{r}\right)$ remains consistent and independent of target. Consequently, under the plane wave approximation, $\sigma \left(k_{r},\;\Omega_{r}\right)$ is the field-free elastic DCS of the target molecule and it contains structural information of the molecule. These tests confirm the validity of the QRS, i.e., the de-coupled extraction of the field-free DCS without dependence on $\theta_{r}$ for various realistic intensities, pulse durations, and target systems.^108,114^ We note that the momentum of the re-scattered electron $k=\left\{k,\theta_{k},\phi_{k}\right\}$ is measured in the laboratory frame with $\theta_{k}=\pi -\theta$ [green arrow in Fig. 1(b); black solid coordinate system], whereas the return momentum $k_{r}=\left\{k_{r},\theta_{r},\phi_{r}\right\}$ (blue arrow) and the scattering angle $\theta_{r}\;$ of the electron are given in the laser polarization frame [Fig. 1(b), dashed coordinate system). Note that the coordinate systems are spherical while the laser's polarization is along the *z* axis. To deduce $k_{r}$ from the measured momentum k, knowledge of the vector potential of the laser field at the time of collision yields the relation $k=-A_{r}+k_{r}$ where the quantities $t_{r}$, $k_{r}$, and $A_{r}$ can be deduced from the SMM, which is well justified for TI and long wavelengths. As explained above, long trajectories dominate the dynamics, this yields $A_{r}=k_{r}/1.26$,^108^ and the IAM further allows to describe the magnitude of the momentum transfer as $q=2k_{r}\text{sin}\left(\theta_{r}/2\right)$. The cross section is composed of two components, incoherent atomic scattering and coherent molecular scattering $\sigma_{\text{tot}}\left(k_{r},\theta_{r}\right)=\sum_{i}{\left|f_{i}\right|}^{2}+\sum_{i\neq j}\;f_{i}f_{j}^{*}e^{iqR_{i,j}}=\sigma_{\text{atom}}+\sigma_{\text{mol}}$. The amplitudes of these contributions vary by almost one order of magnitude. In practice, since the molecular structure information is embedded the coherent superposition, some sort of filtering or fitting is used to subtract the incoherent atomic contribution $\sigma_{\text{atom}}$ from the DCS to extract $\sigma_{\text{mol}}$.

## III. LIED METHODOLOGIES

**FIG. 2.**
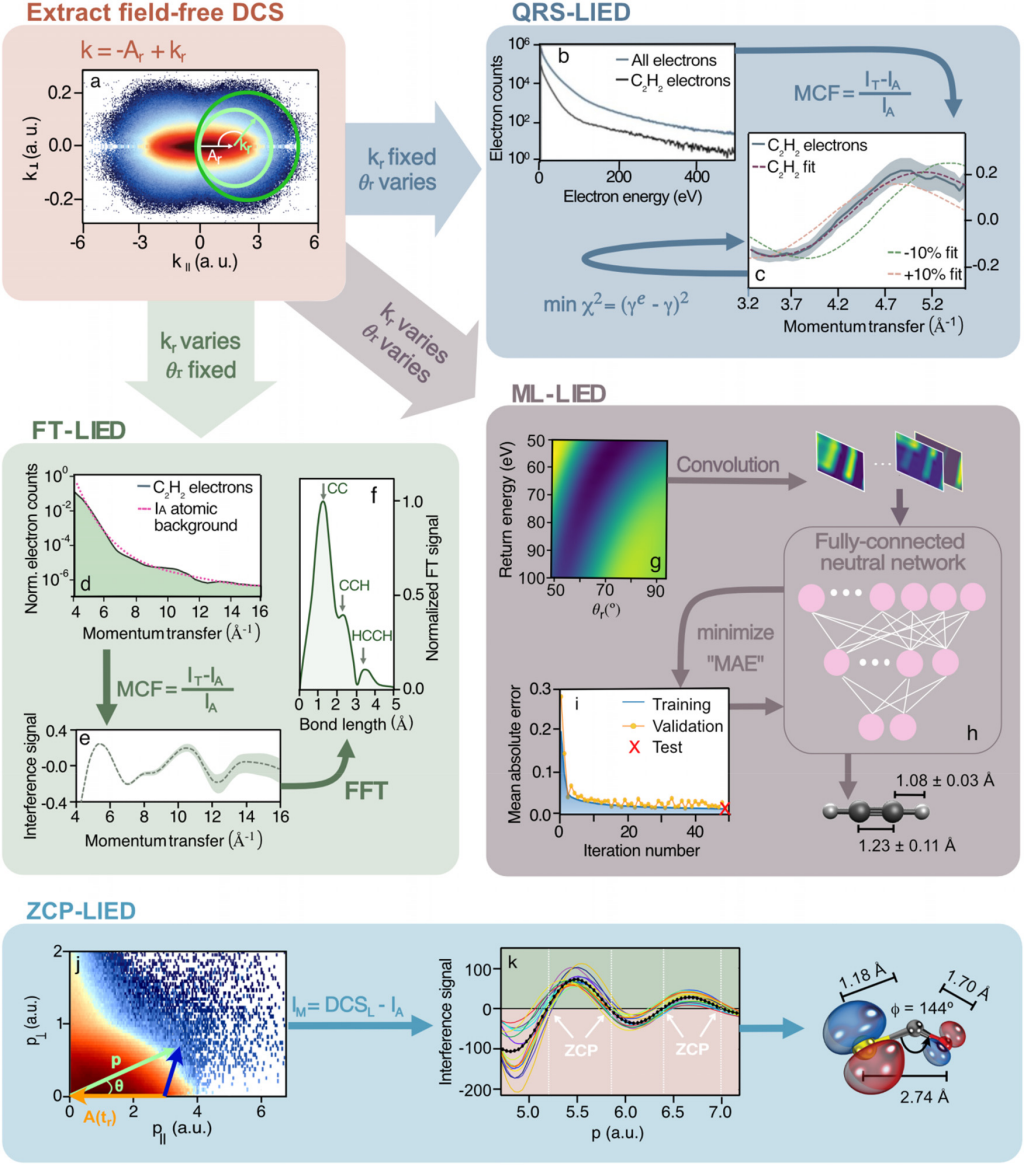
Overview of different retrieval algorithms. QRS-, FT-, and ML-LIED are based on the extraction of field-free DCS in the laser polarization frame (a). By analyzing oscillation embedded in the DCS, the so-called molecular contrast factor (MCF), as a function of momentum transfer for either a fixed rescattering energy (QRS) (b) and (c) or a fixed rescattering angle (FT) (d)–(f), molecular bond lengths are retrieved. The 2D-DCS (g) is the input on the ML algorithms (h) and (i). Data are exemplary shown on C_2_H_2_^+^. ZCP-LIED can extract the bond directly in the scattering frame (j) through analysis of the zero-crossing points (k), and the data retrieval is shown in the example of OCS^+^. More details are found in the text. Figures are adapted from Refs. 34, 41, 86, and 122.

LIED is a table top methodology, which probes a target structure using its own electrons that are predominantly elastically and coherently re-scattered during strong-field induced re-collisions.^7,29,33,36^ Structural information can be extracted when the above-discussed conditions for TI and sufficient impact energy are met and diffraction information is perceptible in the 3D detected momentum distribution as the Fourier transform (FT) in the far-field.^50^ Over the years, different aspects of the physics, such as the necessity for TI and high impact energies,^32,50,115^ aspects of multiple returns,^95^ and alignment and retrieval methods, were investigated. LIED was concomitantly developed from imaging small and linear diatomic molecules like O_2_ and N_2_^32,83,86^ to larger polyatomic systems such as C_2_H_2,_^34^ OCS,^86,116,117^ CS_2,_^38^ NH_3,_^118^ H_2_O,^112^ CF_3_I,^5^ benzene,^119,120^ azo- and chloro-benzene,^121^ and chiral structures like fenchone.^122^ Figure 2 provides an overview over the various implementations that either directly use the 3D DCS (QRS-LIED^32,34,83^), implementations that reduce complexity and achieve high momentum transfer (FT-LIED^41^ or fixed-angle broadband laser-driven electron scattering FABLES^123,124^), or a methodology that tend to issues of complex molecular systems (zero-crossing point (ZCP)-LIED^86^ and machine learning (ML)-LIED^122^).

In 2008, Meckel *et al.*^83^ provided the conceptual ground work of LIED by experimentally showing that recollision leads to diffraction patterns and by detailing the extraction of the field-free molecular DCS. Their laser wavelength of 800 nm at γ=0.6 with U_P_ of 15 eV also brought the previously discussed challenges in extracting bond distances of N_2_ and O_2_ to the fore. In 2012, Blaga *et al.*^32^ conducted another experiment on N_2_ and O_2_ but at much longer wavelengths between 1.7 and 2.3 *μ*m and at γ=0.3 with U_P_ of 70 eV and successfully extracted the bond distances of N_2_ and O_2_ molecules, thus showing that TI conditions are important. In 2015, Pullen *et al.*^34^ showed that LIED can resolve multiple bond distances and image the entire structure of polyatomic C_2_H_2_ without alignment. The experiment provided the first proof that LIED can image hydrogen atoms at 3.2 *μ*m for at γ=0.34 with U_P_ of 50 eV. All seminal papers used the measured DCS and the redundancy provided by the broadband recollision energy spectrum in information extraction; this is the universal method which we call QRS-LIED. While the theoretical concepts of LIED are well established, the exact experimental implementation of LIED can make important differences in achieving molecular structural imaging. For instance, Blaga *et al.* used a dense molecular jet and detected electrons with a time-of-flight (TOF) spectrometer. Data acquisition required rotation of the laser polarization in 5° increments while recording the TOF. While this implementation of QRS-LIED is attractive due to its simplicity, one needs to ensure that single-electron rescattering occurs, i.e., DI or doubly ionized molecular targets or fragmentation does not adversely influence the measurement. The incremental measurement with rotation of the laser polarization can be avoided, as shown by Krečinić *et al.*^125^ with a velocity map imaging (VMI) system since it measures the electronic and ionic kinetic energies and angular distribution with near full 3D collection. Here, a tomographic reconstruction or slicing method is needed to extract the 3D distribution from the 2D measurement.

Another possible caveat with a VMI and TOF measurement are electrons from all atomic and molecular fragments, which are indiscriminately detected from a high-density molecular target. Consequently, their signal contributes to an undesired background, superimposing upon the interference signal of interest. A solution to this problem is coincidence detection. Fragmentation and discrimination are of no issue for small diatomic or triatomic molecules, but it is a tremendous challenge for complex and large molecules, see, e.g., the fragmentation channels displayed in Ref. 34. Addressing this challenge from the onset, we implemented LIED with a reaction microscope (REMI) since cold-target recoil ion momentum spectroscopy (COLTRIMS) provides full 3D kinematic electron–ion detection in coincidence; see Fig. 2 for a sketch. We note that Meckel *et al.*^83^ also used COLTRIMS for their initial measurement on O_2_ and N_2_. COLTRIMS operates with a supersonically expanded molecular jet, which is skimmed to the single-molecule level; thus, it allows measurement of individual molecules without averaging. Furthermore, the 3D detection kinematics of the REMI allows structural reconstruction without alignment or even orientation. Furthermore, multi-coincidence detection discriminates electron and ion fragments; thus, the existence of two or multiple electrons, doubly or multiply charged ions, or fragments is easily detected and is discriminated against.^34^ The specific implementation with a REMI when satisfying TI conditions and core-penetrating collisions achieves the limit of imaging of one molecule with one electron and provides high-quality data without averaging over unwanted effects. Single-shot detection further enables application of post-processing filters based on experimental conditions such as laser intensity, pulse duration, or selection on momentum ranges. Such filters permit discrimination against laser energy fluctuations or drifts by selection of the final kinetic energy of electrons due to the exponential dependence of electron recollision on the laser field amplitude, thus differentiating between rescattering trajectories for multiple cycle duration laser pulses.^86^

### A. QRS-LIED

We term the initial implementation of the LIED concept^7,32,41,119,126^ QRS-LIED to highlight the theory on which it was initially based and to distinguish between the different variations that have been developed for various purposes. Figure 2(a) shows how the field-free DCS is encoded in circles whose circumference relates to a specific rescattering momentum *k_r_* (green arrow). For a given *k_r_*, the circle's origin is shifted by the vector potential $A_{r}$. Under TI conditions, the relation $A_{r}=k_{r}/1.26$ can be used to subtract the field-dependent momentum shift $A_{r}$ from *k_r_* to extract the field-free elastic DCS. Experimentally, in QRS-LIED, rescattering angles ranging from $\theta_{r}=30^{\circ }$ to $140^{\circ }$ are analyzed to achieve sufficient momentum transfer. As a next step, the coherent scattering signal is extracted by subtracting an empirical incoherent and slowly modulating background, or by calculating the incoherent atomic background in case the molecular system is known. To this end, one defines the molecular contrast factor MCF ${=(I}_{T}-I_{A})/I_{A}$, which is as a function of the momentum transfer $q=2k_{r}\text{sin}(\theta_{r}/2)$. The bond lengths are extracted through an iterative process, which compares a theoretically calculated with the experimental MCF.

The QRS-LIED method relies on convergence of a 2D fitting procedure (scattering electron energy and angle), which provides redundancy to improve the quality of the retrieved structural data. Figure 2(c) exemplifies an optimal fitting outcome (violet dashed line) for the retrieval of the structure of C_2_H_2_. Beyond the first demonstrations of Meckel *et al.* and Blaga *et al.* on O_2_ and N_2_, our group successfully retrieved the structure of the larger and polyatomic acetylene molecule (C_2_H_2_) which we were interested in, since the larger system possesses all relevant hallmarks of molecular dynamics such as conical intersections and isomerization pathways. Already our first result was positively surprising since we found that the elastic scattering cross sections for hydrogen at the lower impact energy of 158 eV allowed “seeing” hydrogen atoms.^34^ This is in stark contrast CED energies at which the cross section for hydrogen is essentially zero. The possibility to investigate hydrogen or proton dynamics consequently let to our successful investigation of the de-protonation of C_2_H_2_ and the first imaging of molecular bond breaking.^35^ Having analyzed and retrieved the structure of acetylene with three bonds, leading to 3! = 6 interference features in the DCS due to the factorial scaling, we found that scaling to larger molecules would present a serious challenge for resolving oscillations and finding convergence for peak fitting algorithms. Thus, our conclusion was that QRS-LIED is readily applicable to molecular systems with only a few atoms and a small number of degrees of freedom but challenging to interpret for multiple scattering systems.

### B. FT-LIED

To improve on the fitting and to reach higher momentum transfer and resolution, FT-LIED, also termed FABLES,^123,124^ was developed. FT-LIED^41^ offers a means to directly obtain structural information without prior knowledge of the molecular structure, the need for retrieval algorithms, or *ab initio* simulations. In contrast to QRS-LIED, FT-LIED considers only backscattered electrons, i.e., the signal with maximum momentum transfer. To extract the field-free molecular interference signal from the acquired data, we transform the measured signal by subtracting the vector potential, A_*r*_ from the detected momentum *k* [see Fig. 2(a)]. Through this process, an energy-dependent interference signal, $I_{T}$, is obtained; see Fig. 2(d). To ensure an accurate DCS, achieving a satisfactory signal-to-noise level necessitates integration over a small cone around the backscattering direction. This corresponds to typical values of $\Delta k=0.15k_{r}$. Transferring momentum back into energy, we are interested in the backscattering electrons, i.e., between 2 and $10U_{p}$. We again stress two points. (i) The exact values 2 and $10U_{p}$ originate from the classical recollision model. (ii) To achieve the ultimate attosecond temporal resolution at the limit of LIED, one needs to be mindful of the occurrence of multiple returns and the effects in multi-cycle optical fields. The cleanest solution is restricting the analysis to the range between 8.6 and 10 U_P_. A more practical solution is conducting an error analysis to see how much the temporal range is blurred when including lower U_P_ values. This is our preferred and practical solution. In any case, the field-free molecular interference signal consists of the coherent signal of the molecule $I_{M}$ as well as the incoherent signal from individual atoms $I_{A}$. Next, we subtract the empirical background $I_{A}$ by applying a polynomial fit ( $\sum_{n_{i}=0}c_{i}k_{i}$) as indicated in Fig. 2(d). Here, the fitting coefficients *c_i_* are determined. To avoid overfitting, the order of the polynomial needs to be restricted.^121^ Subsequently, the background is subtracted resulting in a MCF as function of the momentum transfer $q=2k_{r}$; see Fig. 2(e). A Fourier transform yields the distribution of internuclear distances [see Fig. 2(f)]. As in all scattering-based analysis methods, we need to be mindful of the effects of aliasing or unphysical oscillations due to selecting a specific momentum transfer window. We have checked this by analyzing a range of momentum transfer windows and widths to check convergence.^41^ Multi-peak fitting to the so-obtained radial distribution functions retrieves the bond distances. Yet, when the bond lengths are too close to each other, this method struggles to identify the bond distances since peaks overlap and the fitting technique cannot reveal them.

### C. ZCP-LIED

While FT-LIED provides a much simpler analysis procedure and large momentum transfer, ZCP-LIED^86,121^ addresses the general challenge of fitting in scattering methodologies problems by retrieval in the laboratory frame with a simple polynomial background subtraction procedure. We recall that the elastic scattering process takes place in the presence of the laser field, but the measurement occurs in the laboratory frame [see Fig. 1(b)]. For instance, the laboratory-frame angle θ=0° with respect to the laser polarization axis refers to an incident electron that undergoes $\theta_{r}=180{}^{\circ}$ backscattering. In ZCP-LIED, bond lengths are extracted from the positions of the zero-crossing points (ZCP) in the photoelectron spectra, hence the name ZCP-LIED. To obtain the laboratory-frame differential cross section (DCS_L_), scattering events with small angle θ≈ 0° are used for the analysis since they provide efficient momentum transfer [see Fig. 2(j)]. For instance, in Refs. 86 and 121, we chose laboratory scattering angles ranging from θ=0° to 4°, with increments of Δθ=0.25° across the scattering momentum, *p*, for the analysis. We note that ZCP-LIED has the specific advantage that by focusing on zero crossings in the MCF, noise and diminishing amplitude of oscillations have a much-reduced effect on the fitting and data analysis. We obtain the laboratory molecular interference signal ( $I_{M}$) akin to the analysis in the scattering frame, i.e., the background atomic signal $I_{A}$ is subtracted from the DCS_L_. The empirical background is determined from the DCS_L_ through a polynomial function. Figure 2(k) provides an illustrative presentation of the resultant laboratory molecular interference signal ( $I_{M}$) of carbonyl sulfide. The roots of the molecular interference signals define the zero-crossing points (ZCPs); this is indicated by the white arrows. We note that a limited number of ZCPs is sufficient to precisely retrieve molecular structure. For instance, Sanchez *et al.*^86^ demonstrated that merely two ZCPs were adequate to differentiate between the linear and bent OCS^+^ structures. These results demonstrate the method's robustness. Another important aspect of ZCP-LIED is its reduced computational constraints since the analysis fully exploits the broad bandwidth of the imaging electron wave packet for structure retrieval. In comparison with FT-LIED, ZCP-LIED provides greater precision in retrieving internuclear distances, and it addresses the difficulty of distinguishing internuclear distances by further extending the limits of the method.

### C. ML-LIED

Above, we have described the various methodical developments of LIED and the challenge to address the factorial scaling of interference patterns with number of atomic scatterers for larger and complex molecules. The need for further improvement arises when moving from imaging highly symmetric molecular systems like C_60,_ which effectively consist of two different bonds despite the 60 atoms, to complex, asymmetric, and chiral systems like fenchone (C_10_H_16_O), which consist of effectively 19 different bonds. The problem of feature identification is not unique to LIED but a universal challenge for any scattering-based imaging modality. This problem is well known in quantum control in which the problem is to find a global extremum in a multi-dimensional fitting landscape without erroneously converging to a local extremum. Machine learning (ML) methods are ideally suited for pattern recognition and we recently showed^122^ that ML indeed succeeds in retrieving the molecular structure of a molecule as complex as fenchone (C_10_H_16_O). The working principle of the ML algorithm is presented in Figs. 2(g)–2(i). Among the many models for ML, we applied a convolutional neural network (CNN) since it is well-suited for image recognition. The convolution of the 2D-DCS maps with different filters enhances the subtle features of the maps. Numerically, feature maps pass through the fully connected neural network by multiplying the weights between each neuron to predict the atomic position in the molecule [Fig. 2(h)]. The ML algorithm is trained to find the relationship between a molecular structure and its molecular interference signal from the corresponding two-dimensional differential cross-sectional maps 2D-DCS. A database containing thousands of molecular geometries and their DCS is generated to initially train the ML algorithm. The corresponding 2D-DCS map is calculated for each structure using the independent atom model (IAM). The ML algorithm's advantage lies in simultaneously considering multiple degrees of freedom. Its ability to interpolate and learn across the coarse grids of precalculated molecular geometries, i.e., a minimized database, dramatically reduces computational time. The database is then split into training, validation, and test sets to validate the ML model. To evaluate the model's accuracy during training, we define the absolute difference between the predicted and actual atom position (mean absolute error, MAE) as our prediction error [see Fig. 2(i)]. Once the ML model is trained, the experimental 2D-DCS map is used as an input to determine the molecular structure corresponding to the measured interference signal.

We note that any specific implementation of a technique like LIED has its own merits and limitations. Above, we discussed the underlying physics and arising combined requirements for a meaningful implementation of LIED. Clearly, there exist no hard boundaries that delineate a minimally required ponderomotive energy or shortest wavelength, and diffraction data are measured whether these conditions are met or not. However, when the conditions are met, the different implementations of LIED provide a powerful table top attosecond technology to accurately measure molecular structure.

## IV. EXAMPLES OF SINGLE-ELECTRON LIED ON SINGLE GAS-PHASE MOLECULAR STRUCTURES

**FIG. 3.**
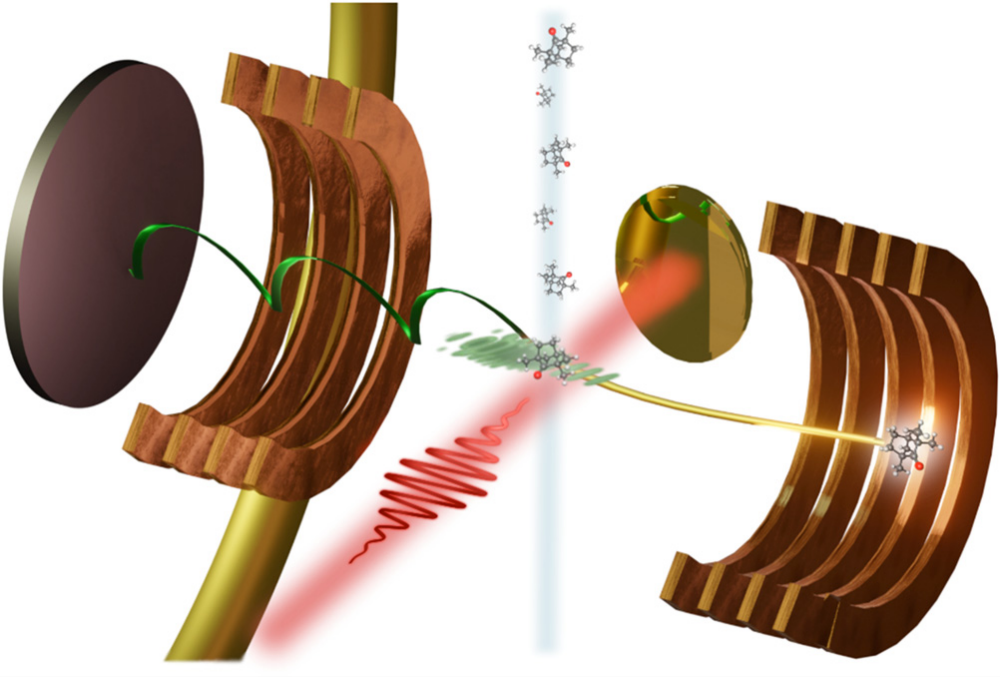
Scheme of a reaction microscope. A supersonic expansion into vacuum leads to molecules whose nuclear degrees of freedoms are essentially frozen. Skimmers reduce the target density such that maximally one molecule is interrogated by one laser pulse. TI leads to liberation of one electron which may scatter of the molecular ion. A magnetic field separated positively from negatively charged particles. A magnetic field is superimposed to increase detection to full 3D by projecting positive and negatively charged to delay line detectors with multi-hit detection capacity. Shown here is the simplest case of single ionization and no fragmentation of the molecular ion. Higher-order effects such as double ionization or multiple electrons are readily detected. Effects such as delayed ionization and fragmentation are also detectable. Fragments can be used to post-orient the molecule without alignment fields. For a comprehensive understanding of REMI's functionality and capabilities, we refer to Refs. 35, 50, and 130.

In the remainder, we like to highlight how the different incarnations of single-molecule LIED with a REMI have allowed us to move from imaging 1D to 2D and to complex and chiral 3D molecular structures. Over the years, we have shown the efficacy of LIED to visualize molecular structures, and, as the complexity and size of molecular systems has increased, we have been developing and improving retrieval methodologies such as FT-LIED, ZCP-LIED, and ML-LIED. As previously outlined, our approach to LIED involves mid-IR pulses,^127–129^ and single-molecule electron–ion coincidence detection in full 3D with a reaction microscope (REMI); see Fig. 3.

The choice of mid-IR wavelengths fulfills the objective of achieving TI at the lowest possible intensity for extracting the field-free DCS. Furthermore, the REMI largely eliminates the need for alignment and avoids any issues related to ensemble averaging. Our investigations have consequently enabled advancements in imaging polyatomic structures in one, two, and three dimensions beyond the confines of diatomic systems.^32,124^ Here, we provide several examples from our work to showcase the utility of LIED. We also refer to the exciting results from other groups using LIED.^32,83,117,120,131^ The success in imaging such a variety of systems also addresses literature in which the authors highlight shortcomings of LIED that arise from implementing the method with near-IR wavelengths, thus, reaching insufficient impact energy and applying the field-free DCS transformation despite operating away from the TI regime. Pushing the technique to the absolute time limit, we have recently shown that two-color control of the LIED field achieves single quantum path selection for a sufficient momentum range, thus addressing previously discussed sub-cycle temporal resolution limitations.^95^

### A. 1D molecular structure retrieval

We start by discussing our group's first experimental result with the acetylene molecular cation^34^ (C_2_H_2_^+^). The experimental implementation with a 3.2 *μ*m laser and intensity of 55 TW/cm^2^ ensured impact energies high enough to measure C–C bond and C–H bond lengths with a resolution of 5 pm, both in aligned and anti-aligned C_2_H_2_ configurations. The measured bond lengths were extracted by applying QRS-LIED^34^ and are in excellent agreement, within 5%, with previously reported acetylene cation distances.^132^ We have also addressed open questions posed in the study by Xu *et al.*,^124^ where the authors managed to retrieve the nitrogen molecule's structure but faced issues with oxygen due to the absence of structural information encoded in the corresponding backscattered electron distribution by FT-LIED; note that similar issues were reported in Ref. 83. The authors of that study concluded that the internuclear axis of interest must be aligned with the laser polarization direction when using the FT-LIED technique to retrieve molecular structure. In contrast, we showed that the problem in retrieval does not originate from the orbital symmetry of the target molecule when carefully applying TI and LIED criteria as described above.^41^ We found that the discrepancy arises from the angular dependence of the ionization probability of the target molecule, which maximizes in parallel with the bond axis for N_2_, but not for O_2_. Not surprisingly, it was found that high-quality molecular alignment reveals the signature of a molecular nodal plane in the backscattering distribution,^116^ but we found this is no impediment to LIED measurements.

### B. 2D and 3D molecular structure retrieval

**FIG. 4.**
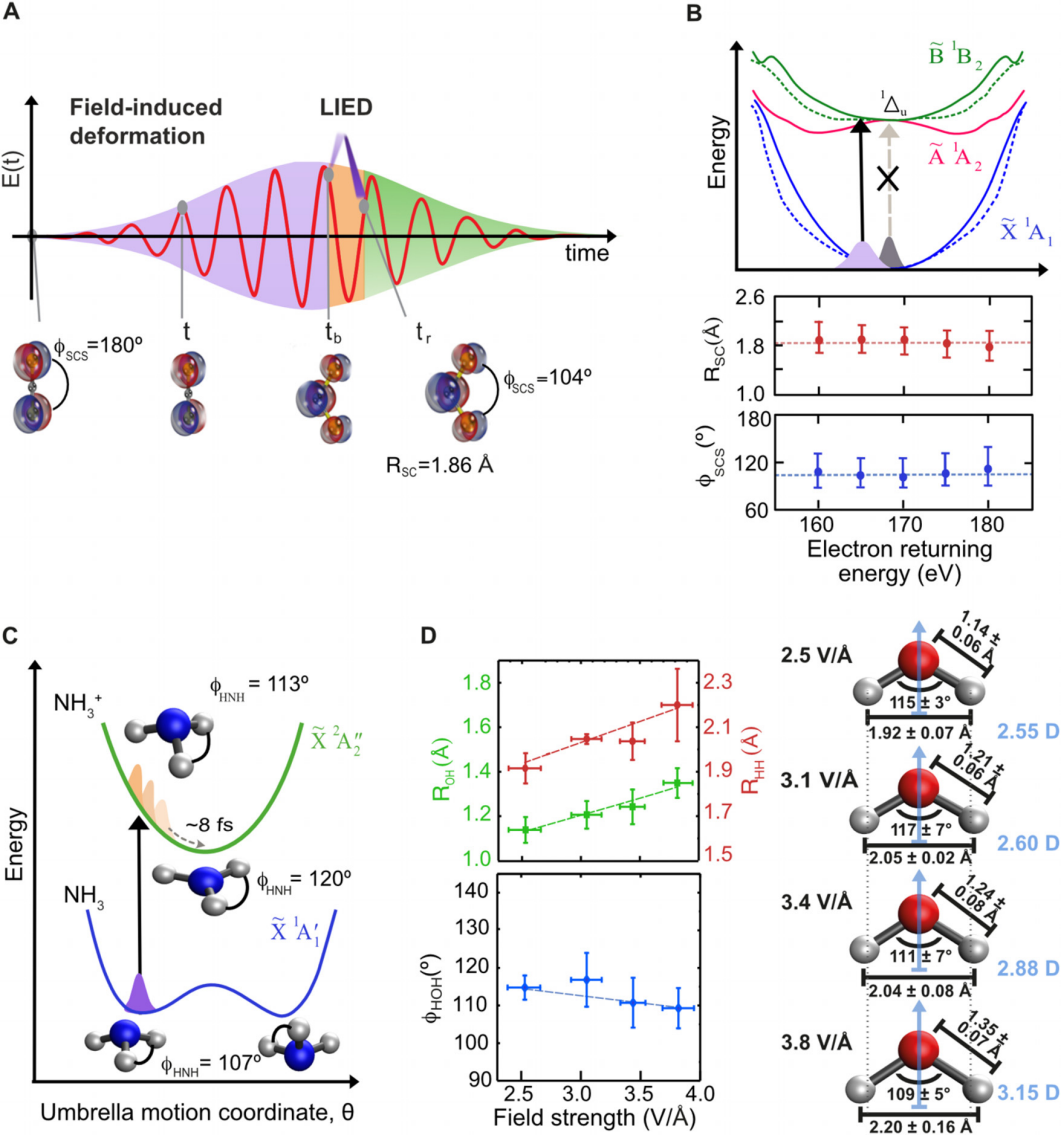
LIED visualizes the dynamics of field-dressed molecules. The strong field acts as a probing mechanism, allowing LIED to capture an instant depiction of the altered molecular structure 7–9 fs after ionization. This concept is exemplified using CS_2_ as an illustration (a). In this manner, LIED provides intricate insights into various physical phenomena, including the Renner–Teller effect observed in CS_2_ (b), the umbrella motion of NH_3_^+^ (c), and the field-induced dipole enhancement through stretching and bending of H_2_O (d). For more details, we refer to the text. Figures (a) and (b) are adapted from Amini *et al.*, Proc. Natl. Acad. Sci. U. S. A. **116**(17), 8173–8177 (2019).^38^ Copyright 2019 National Academy of Sciences. Figure (c) is reproduced with permission from Struct. Dyn. **8**(1), 014301 (2021).^118^ Copyright 2021 AIP Publishing LLC and Figure (d) is adapted from Ref. 112.

In 2016, Wolter *et al.*^35^ captured the breaking of a molecular bond and the deprotonation reaction by imaging the acetylene dication within 9 fs after dissociation with a temporal resolution of 600 as (QRS-LIED). In this study, the dissociation of the molecule was induced by a 6.3-cycle FWHM mid-IR pulse at a peak intensity of 65 TW/cm^2^. The molecule was impulsively aligned, or anti-aligned, using an additional 1.7 *μ*m laser pulse. The LIED experiment succeeded in distinguishing the different kinetic behaviors of the molecule when field-ionized parallel or perpendicular to the laser field while still imaging the entire structure. In the parallel case, the measurement showed that one of the hydrocarbon bonds was heterolytically cleaved with the proton 1.24 Å away from its equilibrium position. The perpendicular case revealed the molecular structure in the quasi-field-free scenario for the dissociative dication. Based on the demonstrated detection sensitivity to hydrogen, we applied FT-LIED to the famous umbrella motion in ammonia (NH_3_).^118^ In equilibrium, the molecule adopts a pyramidal shape (C_*3v*_); see Fig. 4(c). Upon ionization, within roughly 8 fs, NH_3_ undergoes substantial transformation into a planar geometry of D_*3h*_ symmetry.^133^ The longer wavelength with a cycle period of 11 fs provided an ideal testbed for FT-LIED, which allowed us to successfully retrieve the structural geometry of the NH_3_^+^ cation, extracting an H–N–H bond angle of $\phi_{\mathrm{HNH}}=117{}^{\circ}\pm 5{}^{\circ}$ at 7.8–9.8 fs after ionization. This measurement is in excellent agreement with the calculated equilibrium geometry of the field-dressed ground cationic state ( $\phi_{\mathrm{HNH}}^{\mathrm{FD}}=114{}^{\circ}$). Motivated by the possibility of detecting hydrogen and the importance of the solvation shell to study molecular transformations in solution, in 2019 we applied LIED to the water molecule H_2_O.^112^ In the experiment, 97 fs duration pulses were used within varying field strengths ranging from 2.5 to 3.8 V/Å to simulate the salvation shell. Electrons with return kinetic energies ranging from 80 to 460 eV captured the H_2_O structure at return times between 7 and 9 fs following ionization. Employing FT-LIED, we could image the 2D structure of the water cation with varying bond angle; see Fig. 4(d). The measurement simulates the response of the water molecule to various strength of a solvation shell, which mediates structural change by the molecule's dipole. Having measured the effects of symmetry breaking in ammonia, we turned to the Renner–Teller effect,^134^ which is ubiquitous in linear triatomic molecules, leading to bending and stretching, thus changing the geometry from 1D to 2D. An investigation with FT-LIED on CS_2_ leveraged the strong-field dependence of ionization, which is many times perceived as a disadvantage. We showed^38^ that a well-controlled laser pulse with a carefully adjusted peak field can be used to trigger a reaction in the neutral molecule during the rising edge of the pulse before ionization occurs and images its structure; this is depicted in Fig. 4(a). In the absence of the laser field, the transition ${\tilde{X}}^{1}{\Sigma_{g}}^{+}\;\to 1\Delta_{u}$ is dipole forbidden for neutral CS_2_. However, due to the increasing laser field amplitude (85 fs, 90 TW/cm^2^) and non-adiabatic coupling, the molecule undergoes ∼10° bending. The bending lifts the degeneracy of the $1\Delta_{u}$ state into two distinct bent states ${\tilde{A}}^{1}A_{2}$ and ${\tilde{B}}^{1}B_{2}$) of neutral CS_2_. Consequently, transitions between the ${\tilde{X}}^{1}A_{1}$ ground state to the ${\tilde{B}}^{1}B_{2}$ excited state become dipole allowed in the bent geometry [black arrow in Fig. 4(b)]. Consequently, the recollision electron, which is launched at the peak of the pulse envelope, captures the CS_2_ molecule in the excited electronic state ${\tilde{B}}^{1}B_{2}$, which is a bent conformation. We extracted values of $R_{\mathrm{SC}}=1.86\pm 0.23\;Å$ and an S–C–S angle $\phi_{\mathrm{SCS}}=104.0{}^{\circ}\pm 20.2{}^{\circ}$ from QRS-LIED. Note that we have retrieved the structure for varying electron return energy with very similar values [see Fig. 4(b)], thus providing additional confidence and redundancy in retrieving molecular structure.

As a last example for a triatomic molecule, we turned to carbonyl sulfide (OCS). OCS is an atmospherically relevant molecule with ground-state linear confirmation, alike CS_2_. Karamatskos *et al.*^117^ imaged the OCS^+^ structure using QRS-LIED with a 2.0 *μ*m, 38 fs pulse ( $U_{p}=37\;\mathrm{eV}$), and a VMI detection system. The authors observed an indication of stretching of the bond distances of the molecule with $R_{OC}\;=\;1.14\;\pm 0.0\;4\;Å$ and $R_{CS}\;=\;1.55\;\pm \;0.05\;Å$ but that the molecule remains in linear. In contrast, Sanchez *et al.*^86^ measured a notable transition from linear to a bent geometry and explained this with the Renner–Teller effect. ZCP-LIED with a 3.2 *μ*m, 96 fs pulse ( $U_{p}=90\;\mathrm{eV}$) retrieved a significantly bent molecule with O–C–S bond angle of $\phi_{\mathrm{OCS}}=142{}^{\circ}\pm 22{}^{\circ}\;;\;$ see Fig. 3. The discrepancy between the two measurements can be explained by the different laser parameters. Mixed quantum/classical simulations^86^ support our measurement with the $2^{1}A^{\prime }$ excited electronic state of neutral OCS being accessed with the 3.2 *μ*m laser field within 19.5 − 27.0 fs. The observation and theoretical investigation that point at a stretched and bent geometry is also consistent with a previous investigation by Bilalbegović.^135^

### C. 3D complex molecular structure retrieval

Moving to larger molecular structures, in 2016 and 2017, Ito *et al.* used a 1650 nm, 100 fs pulse (I_P_ = 10 eV, U_P_ = 30 eV) with a TOF setup (QRS-LIED) and successfully retrieved the structures of ethylene (C_2_H_4_)^120^ and benzene (C_6_H_6_).^119^ Both systems were retrieved with 5% accuracy and within 10% agreement with the equilibrium geometry. In 2019, Fuest *et al.* used FT-LIED to identify a 6% elongation C_60_ with a 3.6 *μ*m, 100 fs pulse (U_P_ = 97 eV) and based on DFT calculations. However, despite the increase in the number of atoms, these molecules still comprise only two types of bond distances; thus, the retrieval problem was solvable due to the limited number of interferences to be identified in the MCF. Technically, only variations of these two types of bond lengths (C–C and H–C) were considered for structural retrieval, and angles remained fixed, thus severely restricting the solution space. As already explained, the identification of the factorially scaling interference signatures is a major challenge in extracting structural information from a diffraction measurement. To end this, we developed and applied ML-LIED to the fenchone (C_10_H_16_O) molecule which effectively consists of 19 different bonds despite the 27 atoms. To demonstrate the principle and to assess the ML accuracy, we also employed ML-LIED to retrieve the structures of 1D acetylene and 2D carbon disulfide.^122^ The comparison of these results with those obtained through standard fitting routines revealed excellent agreement.

**FIG. 5.**
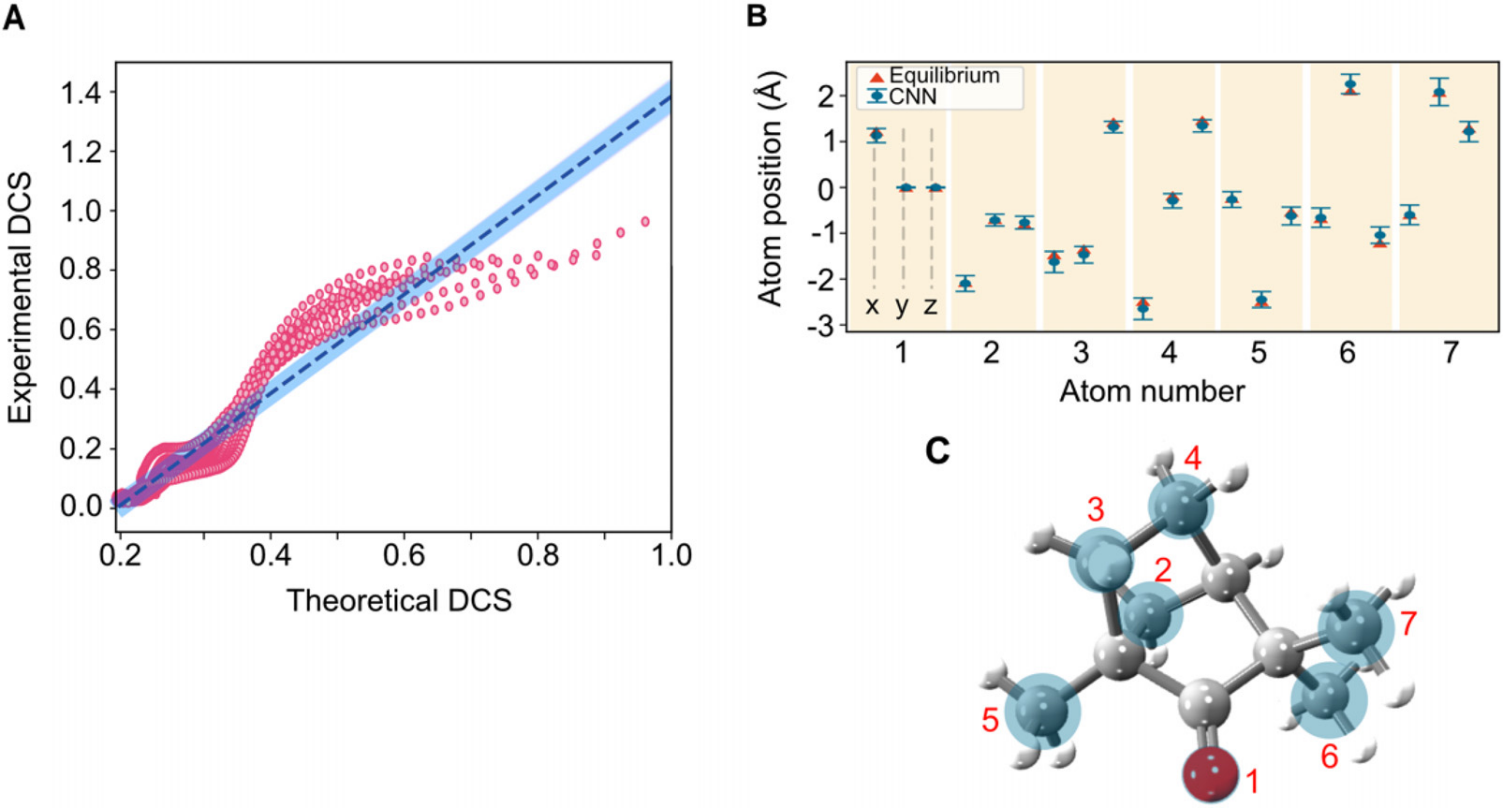
Structure retrieval of the complex molecular structure of fenchone applying ML-LIED. (a) The Pearson correlation coefficient of 0.94 between the experimental and theoretical 2D-DCS calculated based on the ML algorithms' predicted structure shows a strong correlation. The predicted 3D position of seven fenchone (b) atoms compared to the equilibrium structure. Providing a visual representation, the predicted positions with their error bars (blue circles) are overlayed with the 3D molecular structure (c). Adapted with permission from Liu *et al.*, Commun. Chem. **4**(1), 154 (2021).^122^ Copyright 2021 Springer Nature.

In the case of fenchone, ML has the decisive advantage of interpolating and learning between the course grid of precalculated structures and taking into account a manifold of degrees of freedom in the solution space. Thus, we can use a sufficiently reduced database that only considers four groups of atoms of the molecule and a molecule-wide global change in structure. Figure 5(a) shows the mean absolute error for each iteration, achieving a value of 0.02, thus resulting in a strong correlation between the experimental and predicted theoretical DCS with a Pearson correlation coefficient of 0.94. In Fig. 5(b), the 3D Cartesian coordinates of seven atoms within (+)-fenchone are extracted as denoted by blue circles. Notably, the structure of (+)-fenchone obtained through ML-LIED exhibits minor deviations from the equilibrium ground-state neutral molecular structure (depicted as red triangles). The laser field was found to induce these deviations. Figure 5(c) displays the retrieved 3D positions together with the respective errors (blue circles).

The successful retrievals, shown in Fig. 5(b), illustrate that the concept of reducing the computational problem from 27 to a group of 7 atoms permits a way forward to image larger and more complex molecular structures. Such identification is near impossible for complex molecular structures due to the large number of peaks in the radial distribution that overlap due to the multitude of unresolvable two-atom combinations. The reduced computational demands for complex molecules may prove decisive in the application of LIED to time-resolved imaging.

## V. CONCLUSION

We have briefly laid out the basic physics underlying the LIED technique and the conditions required for it to work. The introduction briefly contrasts LIED against conventional electron diffraction for gas-phase molecular imaging before highlighting important aspects for LIED to work, such as fulfilling TI conditions, achieving core-penetrating collisions, and high momentum transfer. We also point out shortcomings, which arise by implementing LIED in violation of these conditions. After a brief historical account of the first implementations, the various incarnations of LIED, ranging from QRS-LIED to FT-LIED, ZCP-LIED, and MP-LIED are placed into context of their development. We then move on to describe experiments which moved LIED from imaging small linear and symmetric diatomic molecules to 2D, 3D, and large complex structures. The specific implementation of our group is single-molecule detection with one electron in full 3D kinematic electron–ion coincidence and we describe why we favored the specific implementation due to recognizing fragments or unwanted background. Overall, we have tried to give an account of the LIED technique until the state of the art. We further briefly point at an experiment on quantum path selection to achieve the ultimate limit in the attosecond temporal range. As every technique, LIED has advantages but also drawbacks and limitations. We hope that our account serves the interested user of LIED by highlighting its implementation and opportunities. Finally, we point at experiments, which have used the intrinsic dynamics of the recollision process to extract frames of molecular dynamics. The next obvious step is to extend the temporal measurement from the narrow window dictated by the recollision time with a true pump-probe measurement. Such an achievement would constitute a long-standing dream of scientists, namely, to achieve single-molecule atomic-resolution images of molecular structural transformation.

## Data Availability

Data sharing is not applicable to this article as no new data were created or analyzed in this study.
